# Bacterial Adaptor Membrane Fusion Proteins and the Structurally Dissimilar Outer Membrane Auxiliary Proteins Have Exchanged Central Domains in *α*-Proteobacteria

**DOI:** 10.1155/2010/589391

**Published:** 2010-04-07

**Authors:** Anthony Y. Xiao, Jing Wang, Milton H. Saier

**Affiliations:** Division of Biological Sciences, University of California at San Diego, La Jolla, CA 92093-0116, USA

## Abstract

Transport systems frequently include auxiliary proteins that perform subfunctions within the transporter protein complex. Two such proteins found in Gram-negative bacteria are the Membrane Fusion Proteins (MFPs) and the Outer Membrane Auxiliary (OMA) proteins. We here demonstrate that OMAs present in *α*-proteobacteria (but not in other bacterial types) contain a long *α*-helical region that is homologous to corresponding regions in the MFPs. The results suggest that during their evolution, OMAs, specifically from *α*-proteobacteria, exchanged their own *α*-helical domain for one derived from an MFP. The structural and functional implications of these findings are discussed.

## 1. Introduction

Transmembrane transport proteins often function in conjunction with auxiliary proteins that facilitate their vectorial activities [1, 2]. Two such auxiliary protein types are found only in the prokaryotic world. These proteins are the Membrane Fusion Proteins (MFPs; Transporter Classification Number (TC#) 8.A.1 in the Transporter Classification Database (TCDB; http://www.tcdb.org/ [3]), which function in conjunction with a variety of transport system types in Gram-negative and Gram-positive bacteria [4] and the Outer Membrane Auxiliary (OMA; 1.B.18) proteins which function in conjunction with a different set of transporter types, exclusively in Gram-negative bacteria [4, 5]. 

MFPs function as “adaptors,” connecting a primary porter in the cytoplasmic membrane, belonging to one of four families of exporters (MFS (TC# 2.A.1), RND (2.A.6), ABC (3.A.1), or AAE (2.A.85)) with an outer membrane factor (OMF; 1.B.17) that provides a porin or channel function in the outer membrane [6]. Thus, in conjunction with an MFP and an OMF, the primary porter in the cytoplasmic membrane pumps molecules out of the cytoplasm, across both membranes of the cell envelope, and into the external milieu without equilibration of solutes in the periplasm, all in a single energy coupled step. Crosslinking studies of the MFP, AcrA of *E. coli *(8.A.1.6.1), with its cognate transporter, AcrB (2.A.6.2.2), and its OMF, TolC (1.B.17.1.1), revealed that AcrA could be crosslinked to both AcrB (via the C-terminal portion of AcrA) and TolC (via the central coiled-coil region of AcrA) [6].

Most MFPs are about 350–500 residues in length and either span the cytoplasmic membrane once at their N-termini or are anchored to the cytoplasmic membrane via a lipoyl moiety. These proteins cluster phylogenetically into subfamilies in accordance with the type of cytoplasmic membrane transporter with which they interact, although the OMFs do not follow this pattern [7]. These transport complexes export a variety of substrates such as toxins, drugs, aromatic acids, peptides, and proteins [5].

The high-resolution 3D structures of two MFPs, MexA of *P. aeruginosa* and AcrA of *E. coli, *have been solved by X-ray crystallography [8, 9]. These proteins consist of three linearly arranged subdomains as suggested by earlier secondary structural predictions [7]. Each of these molecules consists of an N-terminal lipoyl domain, a central 47 Å long alpha-helical coiled-coil hairpin domain, and a C-terminal six-stranded beta-barrel. In the crystal structures, hairpins of neighboring MexA or AcrA monomers pack side by side to form twisted arcs. MFPs may assemble and control conformational channel opening in the complex [6, 8]. They stimulate some but are absolutely required for the functions of other ABC exporters [10, 11]. 

Other transmembrane transporters function together with another type of auxiliary protein that facilitates the export of exo- and capsular polysaccharides. These Outer Membrane Auxiliary proteins (OMAs) comprise a distinct protein family (OMA; 1.B.18) [12]. OMAs are about the same size as MFPs but function with secondary active exporters of the Polysaccharide Transporter (PST) family and primary active exporters of the ABC superfamily (TC #s 2.A.66.2 and 3.A.1, resp.). They are found exclusively in Gram-negative bacteria, and in addition to spanning the periplasm, they form pores in the outer membranes [12]. OMAs are, in general, not homologous to MFPs, and their 3-dimensional X-ray structures show no obvious similarities [8, 9, 13, 14].

The best studied member of the OMA family, WzaK30, has been shown to be required for export of the group 1 K30 capsular polysaccharide in *E. coli* strain E69. Mutations in the encoding gene do not interfere with the synthesis or polymerization of the polysaccharide repeat unit but prevent appearance of the polysaccharide on the cell surface [15]. WzaK30 is a surface-exposed outer membrane lipoprotein, which forms SDS-stable octomeric ring-like structures superficially resembling secretins (1.B.22). Mature WzaK30 is a 359-residue lipoprotein that is synthesized as a precursor with a cleavable 20-residue amino-terminal signal sequence. The thiol group in the processed N-terminal Cys 21 is modified by a thioether-linked diacylglyceryl moiety, and the amino group of Cys21 is acylated. OMAs are believed to form channels through which polysaccharides pass to reach the cell surface. The ring-like homo-octamers (tetramers of dimers) have an outer diameter of ~9 nm and a central cavity of about 2 nm [13, 16]. The native acylated N-terminus is critical for proper assembly. 

The 2.26 Å resolution structure of the 340 kDa octamer of Wza has been reported by Dong et al. [14]. The bulk of the Wza structure is located in the periplasm and comprises three novel domains forming a large central cavity. The revealed Wza structure is open to the extracellular environment but closed to the periplasm. The route and mechanism of capsular polysaccharide translocation have been proposed [14]. Except for the central *α*-helical domains, OMAs and MFPs lack significant structural similarity.

 Another member of the OMA family, KpsD of *E. coli*, has been reported to distribute between the periplasm and the two membranes [17]. Such a possibility is not inconsistent with its role as an outer membrane porin. Together with the primary transporter, KpsE, it was suggested to facilitate transport across the periplasm as well as the outer membrane.

McNulty et al. [18] reported that KdpD is an outer membrane protein involved in the export of group 2 capsular polysaccharides across this membrane. Interestingly, KdpD, KdpE, and the biosynthetic complex comprise a metabolon that is located at the cell poles [18]. The large RhsA protein, previously of unknown function, is a component of this complex and is required for normal polysaccharide export. 

Recently, Cuthbertson et al. [19] have reviewed the roles of OMAs in polysaccharide export. They have suggested changing the name of the family to OPX (outer membrane polysaccharide exporters). They have performed useful phylogenetic analyses, contributing insight into the distribution and structural relationships of these proteins [19].

## 2. Results and Computational Methods Used

**Figure 1:**
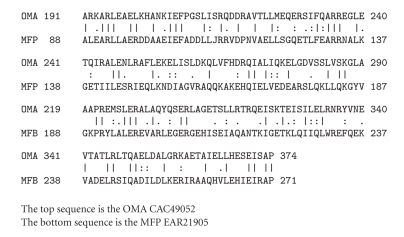
Alignment of a large portion of an OMA from *Sinorhizobium meliloti *(an *α*-proteobacterium) (top; CAC49052) with a homologous portion of an MFP from *Nitrococcus mobilis* (a *γ*-proteobacterium) (bottom; EAR21905). Only OMA homologues from *∞*-proteobacteria were retrieved as top hits when this region of the OMA of *S. meliloti* was used as the query sequence in BLAST searches, while only MFP homologues were retrieved as top hits when this portion of the MFP of *N. mobilis* was used as the query sequence. All proteins retrieved in BLAST searches as top hits that were not from *α*-proteobacteria proved to be MFPs rather than OMAs when this region of the *S. meliloti* homologue was used as the query sequence. For the comparison shown, the GAP program with default settings and 500 random shuffles gave 30% identity, 41% similarity, 0% gaps, and a comparison score of 23 SD. These values are far in excess of the values needed to establish homology [3, 5, 20]. The regions of striking identity are between resides 191 and 374 in the OMA and between residues 88 and 271 in the MFP as shown. These are the *α*-helical regions of both proteins as indicated in Figure 3. Vertical lines, identities; double and single dots, close and distant similarities as defined by the GAP program, respectively. Numbers at the beginning and end of each line of the two aligned sequences refer to residue numbers in the proteins.

Figure 1 shows an alignment of a large portion of an OMA homologue, an OMA-like protein from *Sinorhizobium meliloti, *with the corresponding region of an MFP protein from *Nitrococcus mobilis*. This alignment displays 30% identity and 41% similarity with no gaps and a comparison score based on the GAP program [21] of 23 standard deviations (S.D.). This value is far in excess of what is required to establish homology. 

Using similar criteria, both of these proteins proved to be homologous to established members of their respective families in the Transporter Classification Database (TCDB) [3, 5, 22] as well as in the nonredundant NCBI protein database. Thus, the OMA homologue of *S. meliloti,* used to establish homology with the MFP of *N. mobilis*, is homologous to the established OMA, ExoF of *S. meliloti* (1.B.18.1.1; 34% identity, 55% similarity, and a comparison score of 87 S.D.), while the MFP of *N. mobilis*, used in the same comparison, is homologous to the MFP HlyD of *E. coli* (TC# 8.A.1.3.1; 26% identity, 45% similarity with a comparison score of 53 S.D.). 

**Figure 2:**
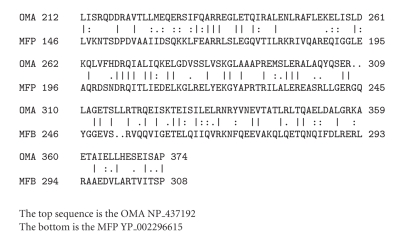
A second alignment, comparable to that shown in Figure 1 using two different proteins that showed similar degrees of similarity throughout the lengths of their *α*-helical regions. The OMA homologue was from *Sinorhizobium meliloti* (top; NP_437192) while the MPF was from another *α*-proteobacterium, *Rhodospirillum centenum* (bottom; YP_002296615). The alignment and comparison scores were determined as for Figure 1 giving 32% identity, 45% similarity, 2 gaps, and a comparison score of 23 S.D.

To confirm this conclusion of homology, we aligned dozens of *α*-helical regions of *α*-proteobacterial OMAs with the corresponding regions of dozens of MFPs. Most of these gave comparison scores in excess of 10 S.D. Figure 2 shows the results of a second example obtained between a different *α*-proteobacterial OMA with an *α*-proteobacterial MFP. Although the sequences are very different from those shown in Figure 1, this alignment also gave a comparison score of 23 S.D., again far in excess of what is required to establish homology.

**Figure 3:**
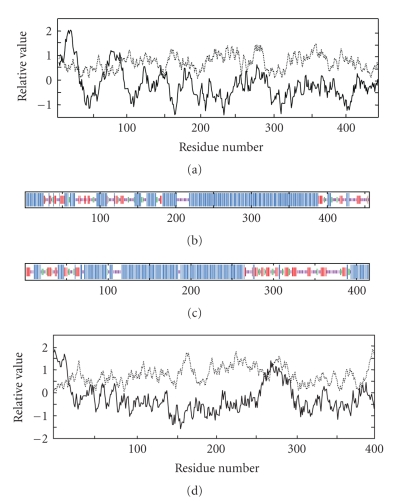
Comparison of the predicted properties of the OMA of *S. meliloti* ((a) and (b)) with the MFP of *N. mobilis* ((c) and (d)) (see Figure 1). (a) Average hydropathy (solid line) and amphipathicity (dotted line) plots for the OMA (WHAT program; [23]). (b) Secondary structure predictions for the same OMA (SOPMA program, [24]). (c) Secondary structure predictions for the MFP of *N. mobilis*. (d) Average hydropathy and amphipathicity plots for the same MFP. The two sequences were aligned using the GAP program ([21]; Figure 1) which was used to prove homology. The single peaks of hydrophobicity correspond to the N-terminal transmembrane signal helices present in both proteins. In (b) and (c), long vertical lines indicate regions of predicted *α*-helix, intermediate length vertical lines indicate regions of *β*-sheet, and shorter vertical lines indicate regions of random coil or *β*-turn.

 The results summarized above establish that central regions of MFPs are homologous to the corresponding domains in a restricted group of OMAs, those present in *α*-proteobacteria. Surprisingly, the sequence similarity with MFPs was not observed in the OMAs from other Gram-negative proteobacteria. We therefore analyzed these proteins further as shown in Figures 3(a)–3(d). The hydropathy plots for the OMA (a) and MFP (d) proteins are shown, as are secondary structure predictions (b) and (c) generated with the WHAT program [23] and the SOPMA program [24], respectively. The similarities between the hydropathy plots and secondary structure predictions are noteworthy. Specifically, we see N-terminal regions in both proteins, which show predicted mixtures of *α*- and *β*-structure followed by central regions, which are entirely *α*-helical. Following these helical regions, we again observe regions predicted by the SOPMA program to contain mixtures of *α*- and *β*-structure with a predominance of *β*-structure.

## 3. Discussion

The three-dimensional structures of representative MFPs and OMAs are surprisingly different [8, 9, 14]. In fact, these structures are so different that no parallels were noted by the investigators conducting the X-ray crystallographic studies. Our results lead to the suggestion that the portions of these proteins that include the central *α*-helical regions, evolved from a common ancestral sequence. Indeed, the portions aligned in Figures 1 and 2 are the only parts to show significant sequence similarities between the *α*-proteobacterial OMAs and the Gram-negative bacterial MFPs. This, of course, does not prove, but it suggests that other portions of these proteins are nonhomologous.

In Gram-negative bacterial MFPs, of the three regions noted above, the *α*-helical region shows the least sequence similarity when compared with other MFPs [7]. Moreover, in some but not all Gram-positive MFPs, this region is deleted, and these MFPs are consequently much smaller [10]. The most logical explanations for these findings are (1) that the long coiled-coil region of an MFP replaced the corresponding helical region in an OMA, specifically in *α*-proteobacteria, (2) the specific amino acyl sequence of this region is minimally important for function in all Gram-negative bacteria, and (3) in some Gram-positive bacteria, where there is no periplasm and no outer membrane, it is not required at all. 

The substitution event that occurred in *α*-proteobacteria probably occurred late, after the separation of *α*-proteobacteria from other proteobacteria. This would account for the high comparison scores obtained, scores, for example, for the alignments shown in Figures 1 and 2. This suggestion is also in agreement with the observation that of the MFPs, those from *α*-proteobacteria are most similar to the *α*-helical regions of *α*-proteobacterial OMAs, clearly implying that the intragenic transfer event occurred in *α*-proteobacteria. It will be interesting to evaluate the detailed three-dimensional structural differences as well as the functional differences of these *α*-proteobacterial OMA homologues in light of these observations. 

 Of particular interest will be the specific roles of the central *α*-helical regions of the proteins within these two families in executing their various functions, which may be multifaceted. Our observations illustrate how poorly understood the structures of diverse members of a protein family can be, even after eucidation of high resolution protein structures for representative members of this family. They also provide unusual and unexpected examples concerning the progression of protein structural divergence during the evolutionary process. Thus, the molecular basis underlying the differences and similarities between the various MFPs and OMA proteins should provide intriguing material for future studies.

## References

[1] Saier MH, Tam R, Reizer A, Reizer J (1994). Two novel families of bacterial membrane proteins concerned with nodulation, cell division and transport. *Molecular Microbiology*.

[2] Huang X, Yan A, Zhang X, Xu Y (2006). Identification and characterization of a putative ABC transporter PltHIJKN required for pyoluteorin production in Pseudomonas sp. M18. *Gene*.

[3] Saier MH, Yen MR, Noto K, Tamang DG, Elkan C (2009). The Transporter Classification Database: recent advances. *Nucleic Acids Research*.

[4] Paulsen IT, Park JH, Choi PS, Saier MH (1997). A family of Gram-negative bacterial outer membrane factors that function in the export of proteins, carbohydrates, drugs and heavy metals from gram-negative bacteria. *FEMS Microbiology Letters*.

[5] Saier MH (2000). A functional-phylogenetic classification system for transmembrane solute transporters. *Microbiology and Molecular Biology Reviews*.

[6] Touzé T, Eswaran J, Bokma E, Koronakis E, Hughes C, Koronakis V (2004). Interactions underlying assembly of the Escherichia coli AcrAB-TolC multidrug efflux system. *Molecular Microbiology*.

[7] Dinh T, Paulsen IT, Saier MH (1994). A family of extracytoplasmic proteins that allow transport of large molecules across the outer membranes of gram-negative bacteria. *Journal of Bacteriology*.

[8] Higgins MK, Bokma E, Koronakis E, Hughes C, Koronakis V (2004). Structure of the periplasmic component of a bacterial drug efflux pump. *Proceedings of the National Academy of Sciences of the United States of America*.

[9] Mikolosko J, Bobyk K, Zgurskaya HI, Ghosh P (2006). Conformational flexibility in the multidrug efflux system protein AcrA. *Structure*.

[10] Harley KT, Djordjevic GM, Tseng T-T, Saier MH (2000). Membrane-fusion protein homologues in gram-positive bacteria. *Molecular Microbiology*.

[11] Tikhonova EB, Devroy VK, Lau SY, Zgurskaya HI (2007). Reconstitution of the Escherichia coli macrolide transporter: the periplasmic membrane fusion protein MacA stimulates the ATPase activity of MacB. *Molecular Microbiology*.

[12] Paulsen IT, Beness AM, Saier MH (1997). Computer-based analyses of the protein constituents of transport systems catalysing export of complex carbohydrates in bacteria. *Microbiology*.

[13] Beis K, Collins RF, Ford RC, Kamis AB, Whitfield C, Naismith JH (2004). Three-dimensional structure of Wza, the protein required for translocation of group 1 capsular polysaccharide across the outer membrane of Escherichia coli. *Journal of Biological Chemistry*.

[14] Dong C, Beis K, Nesper J (2006). Wza the translocon for E. coli capsular polysaccharides defines a new class of membrane protein. *Nature*.

[15] Drummelsmith J, Whitfield C (2000). Translocation of group 1 capsular polysaccharide to the surface of Escherichia coli requires a multimeric complex in the outer membrane. *EMBO Journal*.

[16] Nesper J, Hill CMD, Paiment A (2003). Translocation of group 1 capsular polysaccharide in Escherichia coli serotype K30: structural and functional analysis of the outer membrane lipoprotein Wza. *Journal of Biological Chemistry*.

[17] Arrecubieta C, Hammarton TC, Barrett B (2001). The transport of group 2 capsular polysaccharides across the periplasmic space in Escherichia coli. Roles for the KpsE and KpsD proteins. *Journal of Biological Chemistry*.

[18] McNulty C, Thompson J, Barrett B, Lord L, Andersen C, Roberts IS (2006). The cell surface expression of group 2 capsular polysaccharides in Escherichia coli: the role of KpsD, RhsA and a multi-protein complex at the pole of the cell. *Molecular Microbiology*.

[19] Cuthbertson L, Mainprize IL, Naismith JH, Whitfield C (2009). Pivotal roles of the outer membrane polysaccharide export and polysaccharide copolymerase protein families in export of extracellular polysaccharides in gram-negative bacteria. *Microbiology and Molecular Biology Reviews*.

[20] Saier MH (1994). Computer-aided analyses of transport protein sequences: gleaning evidence concerning function, structure, biogenesis, and evolution. *Microbiological Reviews*.

[21] Devereux J, Haeberli P, Smithies O (1984). A comprehensive set of sequence analysis programs for the VAX. *Nucleic Acids Research*.

[22] Saier MH, Tran CV, Barabote RD (2006). TCDB: the Transporter Classification Database for membrane transport protein analyses and information. *Nucleic Acids Research*.

[23] Zhai Y, Saier MH (2001). A web-based program (WHAT) for the simultaneous prediction of hydropathy, amphipathicity, secondary structure and transmembrane topology for a single protein sequence. *Journal of Molecular Microbiology and Biotechnology*.

[24] Combet C, Blanchet C, Geourjon C, Deléage G (2000). NPS@: network protein sequence analysis. *Trends in Biochemical Sciences*.

